# Interactions between egg storage duration and broiler breeder age on egg fat content, chicken organ weights, and growth performance

**DOI:** 10.1016/j.psj.2020.06.010

**Published:** 2020-06-23

**Authors:** Hedia Nasri, Henry van den Brand, Taha Najjar, Moncef Bouzouaia

**Affiliations:** ∗ Department of Animal Production, National Agronomic Institute of Tunisia, Tunis 1082, Tunisia; † Adaptation Physiology Group, Department of Animal Sciences, Wageningen University, 6700 AH, Wageningen, The Netherlands; ‡ National School of Veterinary Medicine, University of Manouba, Ariana, Sidi Thabet 2020, Tunisia

**Keywords:** breeder age, broiler, chicken quality, egg storage, performance

## Abstract

Egg storage and breeder age are between the most important factors affecting egg lipids, chicken quality, and posthatch performance. To evaluate these factors, including their interaction, the impact of egg storage duration (5, 12, and 19 D), and breeder age (47 and 67 wk) was investigated in Arbor Acres broiler eggs and chickens. Total yolk fat content, chicken organ development at hatch and at 6 D of age, and posthatch performance (at 7 D and 35 D of age) were determined. Total fat content in fresh yolk was lower in 12 and 19 D stored eggs than in 5 D stored eggs (Δ = −2.42% on average). In hatchlings, the heart percentage was not affected by storage duration in the younger flock but was higher after 19 D than after 5 and 12 D of storage in the old flock (Δ = +0.09% on average). Residual yolk weight was higher after 12 D egg storage than after 5 D egg storage (Δ = +1.7 g), with 19 D egg storage in between. Liver and intestine percentage decreased with storage duration. Residual yolk weight (Δ = +1.09 g) and liver percentage (Δ = +0.18%) were higher in old breeders than in younger breeders. At day 6, chicken BW, yolk free body mass, liver percentage, and intestine percentage interacted between egg storage duration and breeder age with the strongest effects in chickens from older breeder after 19 D of storage. Heart percentage was lower after 19 D compared with 5 and 12 D of storage (Δ = −0.05% on average). Feed intake and feed conversion ratio were higher between day 0 to 7 and 0 to 35 after 19 D than after 5 D egg storage (Δ19-5 D = +12 g and +199 g; +0.11 points and +0.09 points, respectively). It can be concluded that when it is needed, eggs from younger breeders should be stored for a prolonged period (≥12 D) rather than those from older breeders.

## Introduction

The quality of the day-old chicken is important for a good start and growth performance of broilers (Meijerhof, 2009). The day-old chicken quality is related to egg quality, and this is in turn affected by, for example, egg storage duration and breeder age (Decuypere and Bruggeman, 2007; Vargas et al., 2009). The egg yolk consists of approximately 50% water, 15% protein, 33% fat, and less than 1% carbohydrates, but the exact composition varies with, for example, breeder age and egg storage duration (Shenstone, 1968; O'Sullivan et al., 1991; Vieira and Moran, 1998; Botsoglou et al. 2012; Qiu et al. 2012; Ren et al. 2013). With the increase of storage duration, egg yolk lipids are susceptible to oxidation, because of a relatively high concentration of polyunsaturated fatty acids (Abreu et al. 2014). With the increase of breeder age, the yolk weight increases because of 2 main reasons: 1) older breeders experience a higher rate of lipoprotein synthesis and deposition (Bray, 1967; Sell et al., 1987) than younger breeders, resulting in larger eggs with larger yolks and 2) with the increase of breeder age, the ovulation interval increases. With longer intervals, the same amount of yolk from hepatic synthesis is deposited in a lower number of oocytes (Zakaria et al., 1983), resulting in larger yolk sizes.

Effects of egg storage duration and breeder age on hatchling quality are shown as well. Goliomytis et al. (2015) reported a decrease of hatchling weight with an increase of egg storage duration. This might be related to the higher water loss during storage (Goliomytis et al., 2015) and/or a direct effect on embryonic growth and development (Christensen et al., 2002). Effects of breeder age on hatchling quality are frequently demonstrated. For example, Iqbal et al. (2016) noted an increase of both hatchling weight and hatchling yield (hatchling weight relative to egg weight) with an increase in breeder age from 30 to 60 wk. Additionally, Nangsuay et al. (2013) reported a higher dry yolk-free body weight of hatchlings from older breeders (53 vs. 29 wk). These findings can probably be explained by the higher energy content in eggs from older breeder hens, especially through the larger yolk size (Nangsuay et al., 2013).

Storage duration and breeder age also affect posthatch performance. Tona et al. (2003) reported a decrease in relative growth, and Petek and Dikmen (2006) noted an increase of the feed conversion ratio (**FCR**) with an increase of egg storage duration. However, other authors (Petek et al., 2003; Goliomytis et al., 2015; Okur et al., 2018) reported a lack of egg storage duration effects on posthatch performances. Concerning the effect of breeder age, Ipek and Sözcü (2015) reported a higher body weight at 7 D of age with younger flocks (33 vs. 62 wk) and explained this result by the higher yolk sac absorption and the advanced intestinal development in hatchlings of the young flock. However, Sabry et al. (2015) noted a higher body weight at 35 D of chicken age with older breeders (49 vs. 32 wk). This difference was explained by the higher chicken weight at hatch in older breeder flocks (Maiorka et al., 2004).

These results suggest that both egg storage duration and breeder flock age affect egg quality, hatchling quality, and later life performance. However, Tona et al. (2004) suggested that neither storage duration nor breeder age has an individual effect. These factors are probably interacting with each other to influence hatchling and chicken variables, which also might explain the ambiguous effects of storage duration and breeder age demonstrated in literature. However, the number of studies investigating this interaction is limited. The objective of the current experiment was to investigate influences of storage duration and broiler breeder age and their interaction on yolk fat content, chicken quality, and posthatch performance.

## Materials and methods

### Experimental Design

The experiment was set up as a 3 × 2 factorial arrangement with 3 egg storage durations (5, 12, and 19 D) and 2 broiler breeder ages (47 and 67 wk of age). Eggs of these storage durations and breeders ages were incubated together, and measurements were performed on the yolk fat content of eggs, chicken quality, and development at hatching, at 6 D of age and on posthatch performance at day 7 and 35 of age. The experiment was performed at the Poulina hatchery, Saouef, Tunisia, between May and July, 2018. The experimental protocol was approved by the Animal Use and Care Committee of the National School of Veterinary medicine (E.N.M.V) of Tunisia.

### Eggs and Egg Storage

In total, 5,460 eggs (910 eggs per breeder age per storage duration) were collected from 2 commercial breeder flocks (Arbor Acres), reared at the same conditions as described by the breeding guide (Aviagen, 2014). Breeder flock ages were 47 and 67 wk. Eggs for the different storage durations treatments were collected at 5, 12, and 19 D before incubation, respectively. During storage, 5,400 eggs were placed on 36 incubation trays of 150 eggs per tray, which were placed on incubation trolleys. Each tray contained eggs of 1 breeder flock age and 1 storage duration. Eggs were stored in 1 storage room in which the temperature was maintained at 16.5°C and the relative humidity at 70%.

### Total Yolk Fat Analysis

After 5, 12, and 19 D of storage, 10 eggs per breeder flock age were sampled for total yolk fat content determination (60 eggs in total). Eggs were broken, and yolk and albumen were separated. The yolk weights were determined. Thereafter, yolks were dried at 106°C for 12 h. The DM was frozen at −20°C until analysis. Total fat extract was performed by Soxhlet (Shandong, China) (AOAC, 1995). Total fat in yolk was calculated as ([extracted total fat weight/yolk sample weight] ∗ DM percentage in wet yolk) ∗100%.

### Incubation

At the end of storage, 6 trays per breeder flock age per storage duration (36 trays in total) were incubated. Trays were alternatively divided over 2 incubation trolleys with 3 trays per breeder age per trolley. The 3 trays at the bottom and the 3 trays at the top of each trolley were not part of the experiment. The 2 trolleys per storage duration were incubated in a Petersime incubator (BioStreamer, capacity of 57,600 eggs, Zulte, Belgium). Experimental trolleys were placed at both sides of the fan. The incubator was further filled with other eggs (10 trolleys), which were not part of the experiment. All incubators were set at an incubation temperature of 100.3°F (at start of incubation), which declined to 97.9°F at the end of day 17 of incubation. Relative humidity was maintained between 74 and 94%. Carbon dioxide level was maintained between 0.1 and 0.85%. Eggs were turned hourly at an angle of 90^o^.

At 18 D of incubation, all eggs were transferred to hatching crates and moved to hatchers. The same order of trays applied in setters was applied for hatching crates in Petersime hatchers (BioStreamer, capacity of 19,200 eggs). Experimental trolleys were placed as the first trolleys near the fan. The hatcher was further filled with other eggs (2 trolleys), which were not part of the experiment. The hatcher was set at a temperature of 98.6°F (at day 18 of incubation), which declined to 96.0°F at the end of incubation. Relative humidity was maintained between 82 and 89%. Carbon dioxide level was maintained between 0.3 and 0.7%. Hatchability per breeder flock age and storage duration was determined for 10 hatching trays of 150 eggs per treatment by counting the number of all hatched chickens per tray.

### Chicken Quality at Hatching and at 6 D of Age

At hatch and at 6 D of age, 20 chickens per breeder flock age and storage duration were randomly selected. These chickens were sacrificed by cervical dislocation and BW, residual yolk weight, and organ (heart, liver, intestines, and stomach [gizzard plus proventriculus]) weights were determined. Yolk-free body mass (**YFBM**) was calculated as BW minus residual yolk weight. Organs weights were expressed as weights relative to YFBM.

### Rearing Phase

At 510 h after the start of incubation, chickens were collected from the hatcher. From each tray, 75 first grade chickens (chickens without exposed brains, 4 legs, 2 heads, cross-beaks, and external residual yolks) were transferred to a rearing house. In total, 2,700 chickens were selected (450 chickens per storage duration and per breeder age), feather sexed (Aviagen, 2014), and placed into 36 floor pens, with a surface area of 7 m^2^ each, covered by wood shavings as litter. Pens were organized in 2 blocks, each containing chickens of the 6 treatments and 3 repetitions per treatment. Each pen contained 75 chickens of both sexes (50:50 ratio) of 1 breeder age and 1 storage duration. The lighting program consisted of 24 light: 0 h dark from day 1 to day 7 and was gradually decreased to 20 light: 4 h dark by day 16 and thereafter remained constant until day 35. The temperature of the rearing house was 31°C from day 1 to day 3 and was gradually decreased to 22°C by day 25 and remained constant thereafter. All chickens received a crumble starter diet from day 1 to day 14 containing 2,990 kcal/kg ME, 22.5% CP, 1.21% digestible lysine, 0.6% methionine, and 0.9% Ca. From day 15 to day 25, they received a grower diet containing 3,070 kcal/kg ME, 22% CP, 1.15% lysine, 0.58% methionine, and 0.8% Ca. From day 26 onward, they received a finisher diet containing 3,150 kcal/kg, 19.5% CP, 1.05% lysine, 0.53% methionine, and 0.7% Ca. Feed and water were distributed ad libitum.

A number of 20 chickens per pen were weighed and identified at placement with permanent dye in their fluff (renewed around 14 D). Marked chickens were weighed at placement (**day 0**), day 7, and day 35 of age. Feed intake (**FI**) was determined per pen at day 7 and 35 as well. Mortality was monitored daily, and dead chickens were weighed. Daily weight gain (**DWG**) was calculated as (BWj–BWi)/(j-i), with i = the first day of measuring and j = the last day of measuring, in which body weight gain of dead chickens was taken into account. Feed conversion ratio was calculated as (weight of consumed feed/BW gain of the whole pen, based on the average weight of the 20 weighed chickens) for the periods 1 to 7, 7 to 35, and 1 to 35 D and corrected for mortality in the respective periods. Efficiency production index (**EPI**) was calculated as ((100 - mortality rate) ∗ DWG/(10 ∗ FCR)).

### Statistical Analysis

Data were processed using the statistical software SAS, version 9.1 (2004). For each parameter, distribution of means and residuals was examined to verify model assumptions. In case data were not normally distributed, a log transformation was performed. For total yolk fat, hatchability, chicken weight, and organ weights at day 0 and 6, a generalized linear model (GLM) was performed. The experimental unit for yolk fat was the egg; for hatchability, it was the tray; and for chicken weight and organ weights, it was the chicken. The model used for these variables was:

[1] Y = μ + Storage duration + Breeder age + Interaction + e

where Y = dependent variable, μ = overall mean, Storage duration = storage duration (5, 12, or 19 D), Breeder age = Breeder flock age (47 or 67 wk), Interaction = interaction between storage duration and breeder flock age, and e = residual error.

For DWG, FCR, and EPI data, a GLM was performed. The experimental unit was the rearing pen. The model used for these variables was:

[2] Y = μ + Storage duration + Breeder age + Interaction + block + e

where Y = dependent variable, μ = overall mean, Storage duration = storage duration (5, 12, or 19 D), Breeder age = Breeder flock age (47 or 67 wk), Interaction = interaction between storage duration and breeder flock age, block = block (1 or 2), and e = residual error.

For mortality percentage, the experimental unit was the pen. These data were analyzed with a Logistic procedure, using model 2. Data are expressed as Least Square Means ± SEM. Multiple comparisons between treatment groups were performed, using Bonferroni adjustments for multiple comparisons. Significance was based on *P* ≤ 0.05.

## Results

### Total Yolk Fat

No interaction between egg storage duration and breeder age nor a breeder age effect was found for total yolk fat percentage. Total yolk fat percentage was higher in 5 D stored eggs than in 12 and 19 D stored eggs (Δ = −2.42% on average, *P* < 0.001) (Table 1).

**Table 1:** Effects of egg storage duration and broiler breeder age and their interaction on percentage of total fat in wet yolk of eggs just before incubation (LSMeans ± SEM).

Treatment	Total fat in wet yolk, %
Storage duration, D	
5	29.75^a^
12	27.65^b^
19	27.02^b^
SEM	0.20
Breeder age, wk	
47	28.28
67	28.17
SEM	0.14
Storage duration ∗ Breeder age	
2∗47	28.70
2∗67	28.28
5∗47	29.77
5∗67	29.72
12∗47	27.93
12∗67	27.37
19∗47	26.72
19∗67	27.32
SEM	0.46
*P*-values	
Storage duration	<0.001
Breeder age	0.60
Storage duration ∗ Breeder age	0.17

^a,b^LSmeans within a column and factor lacking a common superscript differ (*P* ≤ 0.05).

### Hatchability and Organ Development

Hatchability did not show an interaction between egg storage duration and breeder age (Table 2). A storage duration of 19 D resulted in a lower hatchability than a storage duration of 5 D (Δ = 5.0%, *P* = 0.01), with a storage duration of 12 D in between. The 47-wk breeder flock had a higher hatchability than the 67-wk breeder flock (Δ = 25.8%, *P* < 0.001).

**Table 2:** Effects of egg storage duration and broiler breeder age and their interaction on hatchling body and organs weight (LSMeans ± SEM).

Treatment	Hatchability, %	Chicken weight (g)	Residual yolk weight (g)	YFBM (g) 1	Heart (%) 2	Liver (%) 2	Intestine (%)^2^	Stomach (%)^2^
Storage duration, D								
5	74.2^a^	46.88	6.88^b^	40.00	0.83	3.92^a^	4.01^a^	6.18
12	72.6^a,b^	48.30	8.58^a^	39.71	0.86	3.96^a^	3.77^a,b^	6.10
19	69.2^b^	47.11	7.80^a,b^	39.30	0.87	3.67^b^	3.56^b^	6.06
SEM	1.2	0.57	0.32	0.39	0.01	0.06	0.09	0.09
Breeder age, wk								
47	84.9^a^	46.91	7.21^b^	39.70	0.83	3.76^b^	3.81	6.20
67	59.1^b^	47.94	8.30^a^	39.64	0.87	3.94^a^	3.75	6.02
SEM	0.9	0.46	0.26	0.32	0.01	0.05	0.07	0.08
Storage duration ∗ Breeder age								
5∗47	86.7	46.45	6.48	39.97	0.83^b^	3.77	4.14	6.14
5∗67	61.7	47.31	7.29	40.02	0.82^b^	4.07	3.89	6.22
12∗47	86.2	47.52	7.70	39.82	0.85^a,b^	3.98	3.83	6.33
12∗67	59.0	49.08	9.47	39.61	0.86^a,b^	3.95	3.71	5.86
19∗47	81.7	46.78	7.46	39.32	0.82^b^	3.54	3.47	6.15
19∗67	56.7	47.43	8.15	39.29	0.93^a^	3.80	3.65	5.97
SEM	1.6	0.80	0.45	0.55	0.02	0.08	0.13	0.13
*P*-values								
Storage duration	<0.001	0.18	0.002	0.46	0.08	<0.001	0.003	0.66
Breeder age	0.01	0.13	0.005	0.89	0.03	0.009	0.55	0.09
Storage duration x breeder age	0.78	0.85	0.44	0.98	0.02	0.10	0.26	0.12

^a,b^LSmeans within a column and factor lacking a common superscript differ (*P* ≤ 0.05).

Abbreviation: YFBM, yolk-free body mass.

1 YFBM weight = BW—residual yolk weight.

2 Organ percentage = (organs weight/YFBM)∗100.

Organ development was studied on chickens, firstly at hatch and then at 6 D of chicken age. At hatch, an interaction between egg storage duration and breeder age was found for heart percentage (Table 3). Hatchling heart percentages were similar between breeder ages for 5 and 12 D of storage. However, after 19 D of egg storage, hatchlings from 67 wk breeders had higher heart percentage than hatchlings from 47 wk breeders (Δ = +0.11%, *P* = 0.02). Eggs stored for 12 D resulted in hatchlings with higher residual yolk weight than eggs stored for 5 D (Δ = +1.7 g; *P* = 0.002), with eggs stored for 19 D in between. Eggs stored for 19 D resulted in hatchlings with lower liver percentage (Δ = −0.27% on average, *P* < 0.001) than hatchlings originating from eggs stored for 5 and 12 D. Intestine percentage was lower in hatchlings after 19 D egg storage than after 5 D egg storage (Δ = −0.45%, *P* = 0.003), with 12 D storage in between. An older breeder flock (67 wk) resulted in higher residual yolk weight (Δ = +1.09 g, *P* = 0.005) and liver percentage (Δ = +0.18%, *P* = 0.009) than a younger breeder flock (47 wk).

**Table 3:** Effects of egg storage duration and broiler breeder age and their interaction on body and organs weight of chicken at 6 D of age (LSMeans ± SEM).

Treatment	Chicken weight (g)	Yolk weight (g)	YFBM (g)^1^	Heart (%)^2^	Liver (%)^2^	Intestine (%)^2^	Stomach (%)^2^
Storage duration, D							
5	186.0	0. 30^b^	185.7^a^	0.75^a^	4.97	12.47	6.72
12	181.3	0.32^b^	181.4^a,b^	0.72^a^	5.00	12.62	6.75
19	176.4	0.48^a^	175.9^b^	0.68^b^	4.73	11.58	6.96
SEM	1.9	0.15	1.9	0.01	0.08	0.16	0.14
Breeder age, wk							
47	180.2	0.17^a^	180.1	0.72	4.81	12.53	6.80
67	182.2	0.57^b^	181.9	0.71	4.99	11.91	6.82
SEM	1.6	0.12	1.6	0.01	0.07	0.13	0.12
Storage duration ∗ Breeder age							
5∗47	185.2^a,b^	0.09	185.1^a^	0.74	4.70^b,c^	12.54^a,b^	6.74
5∗67	186.8^a^	0.51	186.3^a^	0.75	5.24^a^	12.41^a,b^	6.71
12∗47	176.5^a,b^	0.17	176.4^a,b,c^	0.74	4.87^a,b,c^	13.27^a^	6.66
12∗67	186.0^a^	0.47	186.4^a^	0.71	5.12^b^	11.97^b,c^	6.83
19∗47	178.9^a,b^	0.24	178.7^a,b,c^	0.69	4.86^a,b,c^	11.79^b^	7.00
19∗67	173.9^b^	0.72	173.2^b^	0.68	4.60^c^	11.36^c^	6.91
SEM	2.7	0.21	2.7	0.01	0.12	0.22	0.20
*P*-values							
Storage duration	0.003	0.002	0.003	<0.001	0.05	<0.001	0.45
Breeder age	0.37	<0.001	0.41	0.47	0.07	<0.001	0.93
Storage duration x breeder age	0.04	0.08	0.03	0.39	0.004	0.03	0.81

^a-c^LSmeans within a column and factor lacking a common superscript differ (*P* ≤ 0.05).

Abbreviation: YFBM, yolk-free body mass.

1 YFBM weight = BW—residual yolk weight.

2 Organ percentage = (organs weight/YFBM)∗100.

In 6-day-old chickens, an interaction between egg storage duration and breeder age was found for BW, YFBM, liver percentage, and intestine percentage. Storage had no effect on BW, YFBM, and liver percentage of chickens originating from the 47 wk breeder flock. However, in chickens originating from the 67 wk breeder flock, BW (Δ = −12.5 g on average, *P* = 0.04) and YFBM (Δ = −13.1 g on average, *P* = 0.03) were lower after 19 D of egg storage than after 5 and 12 D of egg storage, whereas liver percentage decreased with storage duration (Δ19-5 D = −0.64%, *P* = 0.004). For the intestine percentage, no effect of breeder flock age was found in 5 D stored eggs, but in 12 and 19 D stored eggs, the intestine percentage was lower in chickens originating from the 67 wk breeder flock than in chickens originating from 47 wk breeder flock (Δ = −1.30 and −0.43%, respectively; *P* = 0.03). The heart percentage was higher in chickens originating from 5 to 12 D stored eggs than in chickens originating from 19 D stored eggs, regardless of breeder age (Δ = +0.05% on average, *P* < 0.001).

### Posthatch Performance

No interactions between egg storage duration and breeder age were found for posthatch performance at 7 and 35 D of age (Tables 4 and 5, respectively). At 7 D of age, no effect of breeder age was found for any of the performance variables. However, FI (Δ = +12.5 g, *P* = 0.01) as well as FCR (Δ = +0.09, *P* = 0.03) were higher after 19 D of storage compared with 5 D of storage, with 12 D of storage in between. No effect on BW and mortality was found at 7 D of age. At 35 D of age, total FI (Δ = +199 g, *P* = 0.001) as well as the FCR (Δ = +0.09, *P* = 0.008) were higher, and EPI was lower (Δ = −33; *P* = 0.02) in chickens originating from 19 D stored eggs than in eggs originating from 5 D stored eggs, with 12 D stored eggs in between. Total FI from day 0 to 35 was higher in chickens originating from the 67 wk breeder flock than from the 47 wk breeder flock (Δ = +111 g, *P* = 0.05).

**Table 4:** Effects of egg storage duration and broiler breeder age and their interaction on posthatch performances of chicken at 7 D of age (LSMeans ± SEM).

Treatment	Body weight day 7 (g)	ADWG^1^	Feed intake day 0 to 7 (g)	FCR^2^	Mortality day 0 to 7 (%)^3^
Storage duration, D					
5	213	22.5	146^b^	0.93^b^	0.88
12	212	22.4	154^a,b^	0.98^a,b^	0.71
19	209	22.1	158^a^	1.02^a^	1.42
SEM	3	0.4	3	0.02	0.29
Breeder age, wk					
47	210	22.0	153	0.99	1.23
67	213	22.6	153	0.97	0.78
SEM	2	0.3	2	0.02	0.24
Storage duration ∗ Breeder age					
5∗47	213	22.4	145	0.93	0.86
5∗67	213	22.5	147	0.94	0.89
12∗47	210	22.1	154	0.99	1.18
12∗67	214	22.7	154	0.97	0.24
19∗47	206	21.6	160	1.06	1.64
19∗67	213	22.6	157	1.00	1.20
SEM	4	0.5	4	0.03	0.41
*P*-values					
Storage duration	0.58	0.74	0.01	0.03	0.21
Breeder age	0.23	0.21	0.94	0.33	0.19
Storage duration x breeder age	0.63	0.67	0.86	0.56	0.51

1 ADWG = average daily weight gain = (BW day 7 – BW day 0)/7.

2 Feed conversion ratio (FCR) = (weight of consumed feed day 7 to day 0/BW gain day 7 to day 0).

3 (Number of dead chicken during monitored period/number of chickens at the first day)∗100.

**Table 5:** Effects of egg storage duration and broiler breeder age and their interaction on posthatch performances of chicken at 35 D of age (LSMeans ± SEM).

Treatment	Body weight (g)	ADWG^1^	Feed intake day 0 to 35 (g)	FCR^2^	Mortality day 0 to 35 (%) 3	EPI^4^
Storage duration, D						
5	2,165	63.7	2,834^b^	1.31^b^	5.3	461^a^
12	2,187	64.3	2,993^a,b^	1.37^a,b^	4.9	449^a,b^
19	2,161	63.5	3,033^a^	1.40^a^	5.8	428^b^
SEM	11	0.3	47	0.02	0.5	8
Breeder age, wk						
47	2,163	63.6	2,898^a^	1.34	5.6	450
67	2,179	64.1	3,009^b^	1.38	5.1	442
SEM	9	0.3	38	0.02	0.4	6
Storage duration ∗ Breeder age						
5∗47	2,156	63.4	2,762	1.28	5.5	468
5∗67	2,174	63.9	2,905	1.34	5.1	454
12∗47	2,166	63.7	2,949	1.36	5.2	445
12∗67	2,209	65.0	3,037	1.37	4.5	453
19∗47	2,167	63.7	2,981	1.38	5.9	436
19∗67	2,155	63.4	3,084	1.43	5.6	419
SEM	15	0.4	66	0.03	0.7	11
*P*-values						
Storage duration	0.18	0.18	0.001	0.008	0.48	0.02
Breeder age	0.20	0.20	0.05	0.09	0.43	0.40
Storage duration ∗ breeder age	0.21	0.21	0.85	0.68	0.96	0.45

1 ADWG = average daily weight gain = (BW day 35 to BW day 0)/35.

2 Feed conversion ratio (FCR)= (weight of consumed feed day 35 to day 0/BW gain day 35 to day 0).

3 (Number of dead chicken during monitored period/number of chickens at the first day)∗100.

4 Efficiency production index = (100 – mortality rate)∗DWG/(10∗FCR).

## Discussion

### Total Yolk Fat

The yolk fat percentage was lower after 12 and 19 D than after 5 D of egg storage. This is consistent with Wang et al. (2017a), who noted a decrease in total yolk fat after 10 D of storage at a storage temperature of 22°C (Δ = −1.42 g/100 g dry egg yolk) and after 20 D of storage at a storage temperature of 4°C (Δ = −3.14 g/100 g dry egg yolk). During egg storage, yolk lipid components (especially phospholipids) can be hydrolyzed by endogenous enzymes, resulting in free fatty acids, which are the main substrates of lipid peroxidation. Lipid peroxidation can generate hydroperoxides and secondary oxidation products, finally decreasing the yolk lipid content (Wang et al., 2017a). Whether the decrease in yolk fat content or the change in yolk lipid composition might have consequences for embryonic development and hatchling quality will be discussed below.

### Chicken Organs

In hatchlings, an interaction between egg storage duration and breeders age was found for heart percentage. The heart percentage was similar between flock ages after 5 and 12 D of storage. However, after 19 D of storage, the heart percentage in hatchlings from the 67 wk breeder flock was higher than in hatchlings from the 47 wk breeder flock. This is consistent with Christensen et al. (2002), who reported in a 53 wk breeder flock a higher heart weight (Δ = +0.46 g) of hatchlings after 14 D of storage compared with 1 D of storage. However, in a 34 wk breeder flock, the heart weight decreased with storage duration. In the current study, the heart percentage found in the young flock (47 wk) was not affected by storage. The impact of interaction disappeared at 6 D of chicken age and only the effect of storage duration persisted. The heart percentage of 6-day-old chickens was lower after 19 D of storage. It can be hypothesized that the lower heart percentage in chickens of the younger flock after 19 D of storage is related to the embryonic development (morphologically and cellular) before storage. Embryos of older breeders are in a more advance morphological stage at oviposition and have a higher number of cells than embryos from a younger flock (Pokhrel et al., 2018). This makes them less vulnerable for apoptosis and necrosis during prolonged storage (Fasenko et al., 2001; Reijrink et al., 2009), and it can be speculated that apoptosis and necrosis might occur in specific regions of the embryo (Reijrink, 2010), resulting in retarded heart development in prolonged stored embryos of younger flocks.

The hatchling BW and YFBM were not affected by storage duration nor by breeder age. However, the hatchling residual yolk weight was higher after 12 D of storage than after 5 D of storage and also higher in the 67 wk breeder flock than in the 47 wk breeder flock. Sklan et al. (2003) also reported a linear increase of residual yolk weight (r = +0.78) with breeder age. Nangsuay et al. (2011) attributed this increase in residual yolk weight to an increase in egg size. They observed that hatchlings from different flock ages had comparable residual yolk weight (wet and dry basis) when the egg weight was the same. The absence of the storage duration impact on the BW and the YFBM was explained by Lilja and Olsson (1987) by the fact that among avian embryos selected for rapid growth posthatching, organogenesis characterized by preferential growth of supply organs over demand organs. Supply organs were defined as heart, liver, and intestine, whereas demand organs were defined as muscle and blood. At 6 D of age, the residual yolk weight represented only 0.20% on average of the chicken BW and was still higher after prolonged egg storage and in older breeders. However, the chicken BW and YFBM were lower in the older breeder flock, particularly after 19 D of storage, which coincided with the decrease of the intestine percentage as described above. The lower chicken BW and YFBM with prolonged storage might be related to the negative effect of storage on intestinal morphology that makes chickens from prolonged stored eggs less able to absorb carbohydrates and proteins than those from short stored eggs at the end of the hatch window (Yalcin et al., 2016).

The hatchling liver percentage was lower after 19 D than after 5 and 12 D of egg storage, regardless of breeder flock age and was also higher in the old breeder flock than in the younger breeder flock. At 6 D of age, only an effect of breeder age was found after 5 D of storage; whereas, this was not present after 12 and 19 D of storage. Koppenol et al. (2015) noted also a higher liver weight (+0.23 g) and percentage (+0.08%) in hatchlings from older breeder (28 vs. 48 wk), and Maiorka et al. (2004) noted in hatchlings a higher liver weight (+0.13 g) with older breeder (60 vs. 30 wk); this difference disappeared at 7 D of age.

The hatchling intestine percentage decreased with storage duration, regardless of breeder age. However, at 6 D of age, the intestine percentage, that was similar between breeder flocks ages after 5 D of egg storage, was lower in the old breeder flock than in the younger breeder flock after 12 and 19 D of egg storage. Maiorka et al. (2004) noted also the absence of difference between intestine percentages with 7-day-old chickens from nonstored eggs, whereas Yalcin et al. (2016, 2017) reported that a prolonged egg storage duration (14 vs. 3 D) negatively affected intestinal morphology. A larger villus width (+3.4 cm) and area (+6.5∗10^−2^ μm^2^) in the jejunum of hatchlings from eggs stored for 3 D was noted compared with eggs stored for 14 D (Yalcin et al., 2017).

Taken together the results of BW, residual yolk weight, YFBM, and intestine percentage, it can be concluded that the negative impact of prolonged egg storage duration appears to be stronger in chickens from old breeders than in chickens from young or prime breeders. The exact reason for this phenomena is not completely clear but might be related to 1) the increased level of apoptosis and necrosis in embryonic cells during prolonged eggs storage, maybe combined with the advanced morphological development of embryos of older breeder (Pokhrel et al., 2018); 2) the lower yolk fat content or the change in yolk lipid composition as a result of hydrolyzation of yolk lipids during prolonged egg storage (Wang et al., 2017b). The chicken embryo derives more than 90% of its energy requirements from the oxidation of yolk fatty acids (Romanoff, 1960; Noble and Cocchi, 1990; Speake et al., 1998). Variations in the fat content of the yolk could contribute to variation in growth (Washburn, 1990) during embryonic life and the subsequent periods (Menge, 1968); 3) the change in absorption capacity of the yolk sac membrane, maybe because of a change in folding with prolonged storage duration; 4) negative effects of oxidative stress during prolonged storage duration (Bakst et al., 2016). Eggs of older breeders have a higher eggshell conductance (O'Dea et al., 2004) and a lower albumen quality (Lapaõ et al., 1999) than eggs of younger breeders, meaning that oxygen can easily approach the yolk through the eggshell and albumen, resulting in higher level of fat oxidation and development of radicals, although this is to our knowledge not proven yet. It is, however, unknown whether or not and to what extend these mechanisms could play a role.

Owing to one or more of this physiological mechanisms during storage, it can be suggested that embryonic development during incubation is delayed and/or retarded, resulting in for example a lower intestinal development, as was also shown by Ipek and Sözcü (2015) in hatchlings of prolonged stored eggs. As the gastrointestinal tract has a major role in chicken growth during the early posthatch growing period (Palo et al., 1995), the decrease of the BW and the YFBM of chickens from long-stored eggs at 6 D of age can be the result of the intestine percentage decrease, but maybe other mechanisms could play a role as well.

### Posthatch Performance

The FI as well as the FCR were higher in chickens originating from eggs stored for 19 D compared with 5 D, with 12 D egg storage in between, regardless of breeder age. The same tendency was noted at 7-day-old and 35-day-old chickens. Petek and Dikmen (2006) also noted the increase of the FCR with the increase of the storage duration (1.75 vs. 1.88, respectively with 5 and 15 D of storage). This resulted also in a decrease of the EPI with prolonged egg storage duration. The increase of the FCR coincided with the increase of the hatchlings residual weight and the decrease of the intestine percentage. It can be speculated that the intestinal development of chickens at the moment of hatching is related to the intensive proliferation and differentiation of enterocytes between day 6 and 10 of age (Mateo et al., 2004). In case of a retarded intestinal development at hatching after prolonged egg storage duration (19 D), chickens might have more difficulties with digestion and absorption of nutrients during rearing, which is reflected in a higher FCR.

We conclude that egg storage duration hardly interacted with breeder flock age on hatchling quality. However, during the initial rearing period (6 D), a larger negative impact was detectable on chickens from older breeders than on chickens from younger breeders after prolonged egg storage (≥12 D). Therefore, it appears recommendable, in case of prolonged egg storage duration (≥12 D) to store eggs from younger breeders rather than those from older breeders to preserve chicken quality.
